# Expression of nuclear progesterone receptor and progesterone receptor membrane components 1 and 2 in the oviduct of cyclic and pregnant cows during the post-ovulation period

**DOI:** 10.1186/1477-7827-10-76

**Published:** 2012-09-07

**Authors:** Marie Saint-Dizier, Olivier Sandra, Stéphane Ployart, Martine Chebrout, Fabienne Constant

**Affiliations:** 1 INRA, UMR 1198 Biologie du Développement et Reproduction, F-78352, Jouy-en-Josas, France; 2 Université Paris-Est, Ecole Nationale Vétérinaire d’Alfort, UMR 1198, 7 av. du Général-de-Gaulle, F-94704, Maisons-Alfort, France; 3 AgroParisTech, UFR Génétique Elevage Reproduction, 16 rue Claude Bernard, F-75231, Paris CEDEX 05, France

**Keywords:** PR, PGRMC1, PGRMC2, Oviduct, Bovine, Expression

## Abstract

**Background:**

Progesterone (P4) may modulate oviductal functions to promote early embryo development in cattle. In addition to its nuclear receptor (PR), P4 may mediate its actions through P4 receptor membrane component 1 (PGRMC1) and its relative, PGRMC2. Two successive experiments were undertaken to characterise the expression of PR, PGRMC1 and PGRMC2 in the bovine oviduct during the post-ovulation period, and to relate their expression to the presence of an embryo, the proximity of the CL and to the region of the oviduct.

**Methods:**

In the first experiment (Exp. I), whole oviduct sections were collected from Holstein cows at Day 1.5, Day 4 and Day 5 post-ovulation (n = 2 cows per stage). The expression of PR, PGRMC1 and PGRMC2 was studied in the ampulla and isthmus by RT-PCR, western-blot and immunohistochemistry. In Exp. II, oviduct epithelial cells were collected from cyclic and pregnant Charolais cows (n = 4 cows per status) at Day 3.5 post-ovulation and mRNA expression of *PR, PGRMC1* and *PGRMC2* was examined in the ampulla and isthmus by real-time quantitative PCR.

**Results:**

In Exp. I, PR, PGRMC1 and PGRMC2 were expressed in all oviduct samples. PGRMC1 was mainly localised in the luminal epithelium whereas PR and PGRMC2 were localised in the epithelium as well as in the muscle and stroma layers of the oviduct. The expression was primarily nuclear for PR, primarily cytoplasmic for PGRMC1 and both nuclear and cytoplasmic for PGRMC2. In Exp. II, mRNA levels for *PR, PGRMC1* and *PGRMC2* were not affected by either the pregnancy status or the side relative to the CL. However, the expression of *PR* and *PGRMC2* varied significantly with the region of the oviduct: *PR* was more highly expressed in the isthmus whereas *PGRMC2* was more highly expressed in the ampulla.

**Conclusions:**

This is the first evidence of PGRMC2 expression in the bovine oviduct. Our findings suggest that P4 regulates the functions of the bovine oviduct in a region-specific manner and through both classical and non classical pathways during the post-ovulation period.

## Background

Progesterone (P4) plays a key role in the establishment and maintenance of pregnancy in cattle [1]. Several studies have demonstrated a positive association between milk or plasma P4 concentration in the immediate post-insemination period and bovine embryo survival and/or development [2-6]. This positive effect of P4 on embryo survival in inseminated cows was by-passed by transfer into the uterus of lactating Holstein cows of fresh morulas or blastocysts [2], suggesting that P4 may improve early embryo development via changes in the oviductal environment during the first days of pregnancy. Indeed, bovine embryos develop to the morula stage within the oviduct in the first 5 days of pregnancy, before entering the uterus [1]. The proximal part of the oviduct, referred to as the ampulla, is particularly implicated in the transport of the oocyte-cumulus complex and fertilisation, whereas the distal part, named the isthmus, is responsible for sperm storage and embryo transport toward the uterus [7-9]. The oviduct epithelium provides nutrients (e.g. energetic substrates, ions, amino acids) and local growth factors [10,11], that can be modified quantitatively [12] and qualitatively [13] by circulating P4 secreted by the *corpus luteum* (CL). Moreover, P4 could influence embryo development through the regulation of its transport to the uterus [14]. In cattle, P4 has been shown to reduce oviduct motility *in vivo*[15] and to reduce ciliary motility in tubal explants *ex vivo* through rapid non genomic actions [16]. However, the mechanisms by which P4 modulates the tubal micro-environment in cattle are still poorly understood.

P4 may act in the oviduct through at least two types of receptors: the conventional nuclear receptor PR, that exists in two isoforms PRA and PRB (arising from a single gene) which are both expressed in the bovine oviduct [17,18], and two potential P4 receptors, referred to as progesterone receptor membrane component (PGRMC) 1 and the closely related PGRMC2, encoded by two different genes [19]. While minimal information is available concerning PGRMC2, PGRMC1 is a vertebrate 26–28 kDa protein that contains a single membrane-spanning domain and belongs to the membrane-associated P4 receptor (MAPR) family [20]. Recent studies suggest that PGRMC1 mediates several P4 actions in reproductive organs, such as anti-apoptotic action in rat granulosa cells [21], sperm acrosome reaction in porcine and human [22], oocyte maturation in bovine [23,24] and myometrium relaxation during pregnancy in human [25]. Furthermore, PGRMC1 has recently been immuno-localised in the bovine genital tract, including the oviduct, during the oestrous cycle [26].

In order to explore the expression of nuclear and membrane P4 receptors in the bovine oviduct during the post-ovulation period, two experiments were carried out. The first experiment (Exp. I) was undertaken to detect mRNA and localise protein expression of PR, PGRMC1 and PGRMC2 in the bovine oviduct during the first 5 days post-ovulation. The second experiment (Exp. II) was designed to compare the expression of these three receptors in oviduct epithelial cells at Day 3.5 post-ovulation in relation to the pregnancy status, to the side relative to the CL and to the region of the oviduct.

## Methods

### Treatment of animals and oviduct collection

All procedures described within were approved by the Regional Ethical Committee of Paris-Sud and performed in accordance with the International Guiding Principles for Biomedical Research Involving Animals.

In Exp. I, six non lactating Holstein cows (age: 7.0 ± 1.4 years) were synchronised using a subcutaneous implant of P4 (Crestar SO®, Intervet-Schering-Plough, Angers, France) left for 10 days, with 500 μg of cloprostenol (Estrumate®, Intervet-Schering-Plough) IM-injected 2 days before implant removal and 400 IU equine Chorionic Gonadotropin (CHRONO-GEST® PMSG, Intervet-Schering-Plough) IM-injected on the day of implant removal. Day 0 was considered as the day of presumed ovulation, i.e. 48 h after implant removal. Cows were killed either at Day 1.5, Day 4 or Day 5 post-ovulation (n = 2 for each stage) in a local INRA slaughterhouse for tissue collection.

In Exp. II, 20 multiparous non lactating Charolais cows (age: 7.1 ± 0.3 years) were synchronised for oestrus using a subcutaneous implant of P4 (Crestar SO®) inserted for 11 days, with 10.5 μg busereline acetate (Receptal®, Intervet-Schering-Plough) IM-injected on the day of implant insertion, 15 mg luprostiol (Prosolvin®, Virbac, Carros, France) IM-injected 2 days before implant removal and 400 IU equine Chorionic Gonadotropin (CHRONO-GEST® PMSG) IM-injected on the day of implant removal. Day 0 was considered as the day of presumed ovulation, i.e. 48 h after implant removal. In order to get pregnant oviducts, 12 cows out of 20 were artificially inseminated twice at Day 0 and Day 0.5. All cows were killed at Day 3.5 post-ovulation for tissue collection in a local INRA slaughterhouse. In Exp. I and II, ovaries and genital tract were recovered from cows within 20 min of death. Cows that did not ovulate or that did ovulate twice (two corpora lutea) were discarded. Furthermore, the appearance of the CL varied with the post-ovulation stage: a small corpus hemorrhagicum was observed at Day 1.5 post-ovulation, a very early corpus luteum, occasionally filled with blood, was observed at Days 3.5-4 post-ovulation, and a large corpus luteum was observed at Day 5 post-ovulation, as previously described [27]. Oviducts ipsilateral and contralateral to the CL were separated from the uterus at the utero-tubal junction, trimmed free of surrounding tissue then flushed with 2 ml of Phosphate Buffer Saline (PBS, Sigma, Saint-Quentin Fallavier, France). In Exp. II, the flushing from the oviduct ipsilateral to the CL was examined under the microscope for the presence of an oocyte or an embryo. Non inseminated and inseminated cows in which no oocyte and no embryo, respectively, could be recovered, were excluded. Eventually, four cyclic cows (for which one oocyte was found in the oviductal flushing) and four pregnant cows (for which one embryo at the 8- to 16-cell stage was found in the oviductal flushing) were analyzed in Exp. II.

### Preparation of oviduct samples

In Exp. I, ampulla and isthmus were separated then cut into 1-cm long sections and whole tissues were either snap frozen in liquid nitrogen or embedded in OCT (Embedding Matrix, Cellpath, Newton, UK), then frozen in liquid nitrogen vapours and stored at – 80°C pending analysis.

In Exp. II, the ampulla and isthmus were opened longitudinally and oviduct epithelial cells were recovered by scraping the mucosal layer with a sterile scalpel. Cells from both regions were immediately transferred into a sterile cryotube and snap frozen in liquid nitrogen then stored at – 80°C pending analysis.

### Preparation of cDNAs

In Exp. I, total RNA was extracted from whole oviduct sections in TRIzol reagent (Invitrogen, Cergy-Pontoise, France) using a homogeniser (Polytron® PT-MR 2100, Kinematica AG, Littau, Switzerland), then treated with recombinant DNase I (Roche Applied Science, Mannheim, Germany) as described in the manufacturer’s instructions, and stored at −80°C until use in reverse transcription (RT).

In Exp. II, total RNA was extracted from oviduct cells using the RNeasy Mini kit (Qiagen, Courtaboeuf**,** France) then treated with RNase Inhibitor (40 U/μl; Invitrogen) and recombinant DNase I (Roche Applied Science). RNA samples were then migrated on an ethidium bromide-treated 1% agarose gel to assess their integrity (by observation of 18S and 28S rRNA intensities after UV light exposure). Six RNA samples (out of 32) were suboptimal and discarded.

In both studies, RNA samples were quantified by spectrophotometry and 1 μg was reverse transcribed using oligo(dT) primers (Invitrogen) and the Superscript II reverse transcriptase (Invitrogen) in a 20-μl reaction mixture according to manufacturer’s instructions. The reverse-transcribed cDNA was diluted 5 times (100 μl final volume) and stored at – 20°C until used as template in conventional or real-time quantitative PCR mixture.

### Amplification of cDNA by conventional and quantitative real-time PCR (qPCR)

Same primer sets were used in both conventional and qPCR. Primer sets were developed using known bovine sequences and designed using the Primer 3 web interface [28] for target genes and using Primer Express software (v3.0, Applied Biosystems, Life Technologies, Carlsbad, CA, USA) for two reference genes, *GAPDH* and *SLC30A6* (see Table 1 for details). Primers for *PR* were designed to detect both PRA and PRB isoforms. The specificity of each conventional and quantitative PCR products was verified in the Basic Local Alignment Search Tool [29] and by DNA sequencing (Beckman Coulter Genomics, Takeley, UK). Conventional PCR reactions were performed in Exp. I using a Mastercycler (Eppendorf, Hamburg, Germany). Reactions were performed in a 25-μl mixture containing 0.5 IU of Taq polymerase and 5× buffer (QBiogene, Illkirch, France), 0.5 μM of each primer, 10 mM dNTPs, and 1 μl of diluted cDNA. A negative control using water instead of cDNA was added to each PCR reaction. The amplification of ACTB cDNA was used as internal control (primer sequences in Table 1). Reaction times were: 1 min at 94°C then 35 cycles of 30 sec at 94°C, 30 sec at 60°C and 30 sec at 72°C, then 15 min at 72°C. The amplified products were resolved by electrophoresis on an ethidium bromide-treated 2% agarose gel. The quantification of mRNAs was examined in Exp. II by qPCR on a LightCycler® 480 apparatus (Roche, Mannheim, Germany). For each gene, a 10-fold dilution series of the purified PCR product (Wizard PCR Preps DNA Purification System, Promega, Madison WI, USA) was amplified simultaneously with the samples to establish a standard curve. Reactions were performed in a 20-μl reaction mixture containing either 5 μl of diluted cDNA or diluted standard, 10 μl of LightCycler 480 SYBR Green I Master mix (Roche), 0.3 μM of forward and reverse primers and PCR-grade water up to the final volume. As negative control, water instead of cDNA was used. Cycling conditions were: denaturation at 95°C for 10 min followed by 45 cycles of amplification (95°C for 10 sec, 60°C for 10 sec and 72°C for 10 sec) with a single acquisition of fluorescence at the end of the extension step. After amplification, the samples were heated at 0.1°C/sec from 60 to 97°C with continuous reading of fluorescence to obtain a melting curve. The specificity of qPCR product was verified using the melting curve analysis program of the Lightcycler software (LightCycler® 480 Software release, version 1.5.0.33, Roche). All qPCR products displayed a single peak in the melting curve analysis (data not shown). The concentration of each gene was calculated in duplicate by reference to the respective standard curve using the maximum second-derivative analysis of the Lightcycler software (Roche). Finally, the relative level of gene expression was expressed as the ratio of target gene mean value to the geometric mean value of the two reference genes, *GAPDH* and *SLC30A6*.

**Table 1:** Primer sets used in conventional and real-time PCR

**Gene name (abbreviation)**	**GenBank accession no.**	**Primer sequence (5’ → 3’)**	**Product length (bp)**
Progesterone receptor (PR)	AY656812	Forward: GATGCTATATTTTGCGCCTGA	266
		Reverse: CTCCTTTTTGCCTCAAACCA	
Progesterone receptor membrane component 1 (PGRMC1)	BC1184441	Forward: GCAAGCTTTGGCGAAAATCA	121
		Reverse: CCCCTCGCATGTCCAATCAT	
Progesterone receptor membrane component 2 (PGRMC2)	NM_001099060	Forward: AGGGGAAGAACCGTCAGAAT	280
		Reverse: AAGCCCCACCAGACATTACA	
Glyceraldehyde 3-phosphate dehydrogenase (GAPDH)	NM_001034034	Forward: GCTGAGGCTCCCATGTTTGT	151
		Reverse: TCATAAGTCCCTCCACGATGC	
Solute carrier family 30 (zinc transporter), member 6 (SLC30A6)	NM_001075766	Forward**:** TGATGAGGAAACCTAGCCCTGCC	143
		Reverse: TCGGGCTGCTCCAAAAAGCGT	
Beta actin (ACTB)	NM_173979	Forward: CTGGACTTCGAGCAGGAGAT	140
		Reverse: AGGAAGGAAGGCTGGAAGAG	

### Western blot analysis

In order to test the specificity of antibodies raised against P4 receptors, three oviduct sections from Exp. I were used for protein extraction and western-blot analysis. Tissues were homogenized in lysis buffer (Ripa buffer, Pierce, Rockford, USA) containing proteinase and phosphatase inhibitors for protein extraction. Protein samples (60 μg) were separated on a SDS-PAGE gel (Biorad, Marnes-la-Coquette, France) containing 10% acrylamide and 5% β-mercaptoethanol then transferred onto a 0.2-μm nitrocellulose membrane (Schleicher & Schuell, Dassel, Germany) overnight at 4°C. Membranes were blocked with 5% dried milk in Tris Buffer Saline (TBS: Tris–HCl buffer 10 mM pH 8, NaCl 150 mM) for 1 h then incubated with one of the following primary antibodies: 1) a monoclonal mouse anti-PR antibody (MA1-410 raised against a peptide corresponding to amino acids 553–547 of the human PR; Affinity BioReagents, Golden, USA) and diluted at 1:200; 2) a polyclonal rabbit anti-PGRMC1 antibody (raised against a peptide corresponding to amino acids 1–14 of the native porcine PGRMC1 [30]; gift from Prof. M. Wehling, University Medicine Mannheim, University of Heidelberg, Germany) diluted at 1:500; 3) a monoclonal mouse anti-PGRMC2 antibody (clone 3C11 raised against a peptide corresponding to amino acids 124–224 of the human PGRMC2; Sigma-Aldrich, Saint Louis, USA) diluted at 1:200. Loading controls were carried out using either an anti-α-tubulin (mouse monoclonal antibody, clone DM1A, Sigma) or an anti-β-actin (mouse monoclonal antibody, clone AC-74, Sigma) antibody. Membranes were incubated in 2% dried milk in TBS containing 0.05% of Tween-20 (TBS-Tween) overnight at 4°C. After washing in TBS-Tween, the membranes were incubated with either an anti-mouse (for PR and PGRMC2) or an anti-rabbit (for PGRMC1) horseradish peroxidase-conjugated IgG secondary antibody (Sigma) at dilutions of 1:5000 and 1:30.000, respectively, in 2% dried milk in TBS-Tween for 1 h at room temperature. After washing in TBS-Tween, the membrane was incubated with enhanced chemiluminescence reagent detection solution (Super Signal West Pico, Pierce) for 5 min in the dark. Finally, a film (Hyperfilm ECL, Amersham Biosciences, Orsay, France) was exposed to the membrane to visualize protein expression.

### Immunohistochemistry

Frozen oviduct samples from Exp. I were serially sectioned (7 μm) with a cryostat. Sections were mounted on Superfrost Plus slides (Menzel-Gläser, Braunshweig, Germany), fixed for 5 min in acetone at – 20°C then stored at – 80°C until use. Non-specific protein binding was inhibited by incubation with normal porcine serum (1:10 dilution in PBS; Interchim, Montluçon, France) for 30 min at room temperature. Sections were incubated with either the anti-PR antibody (1:1000 dilution in PBS), the anti-PGRMC1 antibody (1:1000 dilution in PBS) or the anti-PGRMC2 antibody (1:200 in PBS). Sections were incubated overnight at 4°C in a moist chamber. After washing in PBS, sections were incubated with either anti-mouse (for PR and PGRMC2) or anti-rabbit (for PGRMC1) biotinylated secondary antibodies (LSAB kit, Dako, Trappes, France) at room temperature for 30 min. Sections were then treated with 3% H_2_O_2_ for 5 min and incubated with a streptavidin-horseradish peroxidase complex (LSAB kit) at room temperature for 15 min. The signal was detected using a solution of 3-amino-9-ethylcarbazole (AEC; LSAB kit) for 10 min. Sections were counterstained with hematoxylin and mounted in Immu-Mount mounting solution (Thermon Electron, Courtaboeuf, France) for light microscopy. For control sections, the primary antibody was replaced at the same dilution by mouse IgG for PR, rabbit serum for PGRMC1 or mouse IgG_1_ for PGRMC2.

### Statistical analysis

Data are presented as means ± SEM. For statistical analysis of qRT-PCR data, the GLM procedure of SAS software (SAS Institute Inc., Cary, NC, USA) was used. A three-way ANOVA was used to study the effect of the cow status (cyclic *versus* pregnant), the oviduct side relative to that of the CL (ipsilateral *versus* contralateral) and the oviduct section (ampulla *versus* isthmus) on mRNA levels, followed, when necessary, by a multiple *t*-test analysis. To account for potential correlations between observations made on the same cow, the factors “oviduct side” and “oviduct section” were considered as repeated measures. A p value < 0.05 was considered significant.

## Results

### Experiment I: expression and localisation of PR, PGRMC1 and PGRMC2 in whole oviduct sections between Day 1.5 and Day 5 post-ovulation

**Figure 1:**
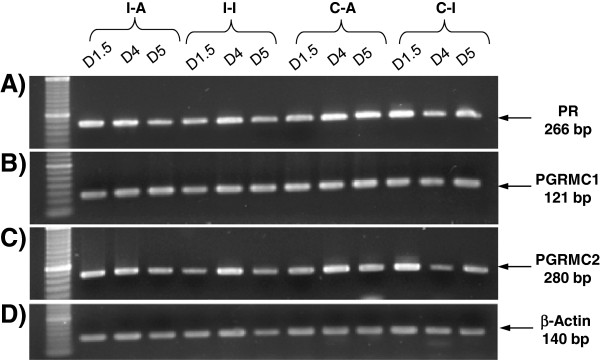
**Expression of PR, PGRMC1 and PGRMC2 mRNAs in the bovine oviduct.** Messenger RNA transcripts for PR (**A**), PGRMC1 (**B**) and PGRMC2 (**C**) detected by conventional RT-PCR in bovine whole oviduct sections at Day 1.5, Day 4 and Day 5 post-ovulation. Experiments were repeated twice and one representative assay is shown. Arrows indicate the size of the amplified product. I-A: ipsilateral ampulla; I-I: ipsilateral isthmus; C-A: contralateral ampulla; C-I: contralateral isthmus.

Conventional RT-PCR evidenced the expression of *PR*, *PGRMC1* and *PGRMC2* mRNAs in both ipsilateral and contralateral ampulla and isthmus at Day 1.5, Day 4 and Day 5 post-ovulation (Figure 1) whereas negative controls using water instead of cDNA showed no signal (data not shown).

**Figure 2:**
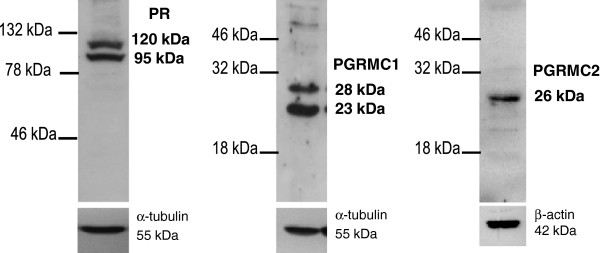
**Western blots for PR, PGRMC1 and PGRMC2 in the bovine oviduct.** Western blot analysis of PR, PGRMC1 and PGRMC2 in one bovine whole ampulla section at Day 5 post-ovulation. Molecular weight markers are indicated on the left of each blot and molecular weights of the bands are indicated on the right. Experiments were repeated three times and one representative blot is shown.

The specificity of antibodies raised against target proteins was examined by western blot analysis. The anti-PR antibody detected both PR-A (95 kDa) and PR-B (120 kDa) isoforms in the ampulla and isthmus at each stage examined (see Figure 2 for one ampulla at Day 5). Western blotting analysis of the same oviduct sample detected two bands at 23 kDa and 28 kDa using the anti-PGRMC1 antibody, and a single band at 26 kDa using the anti-PGRMC2 antibody (Figure 2).

**Figure 3:**
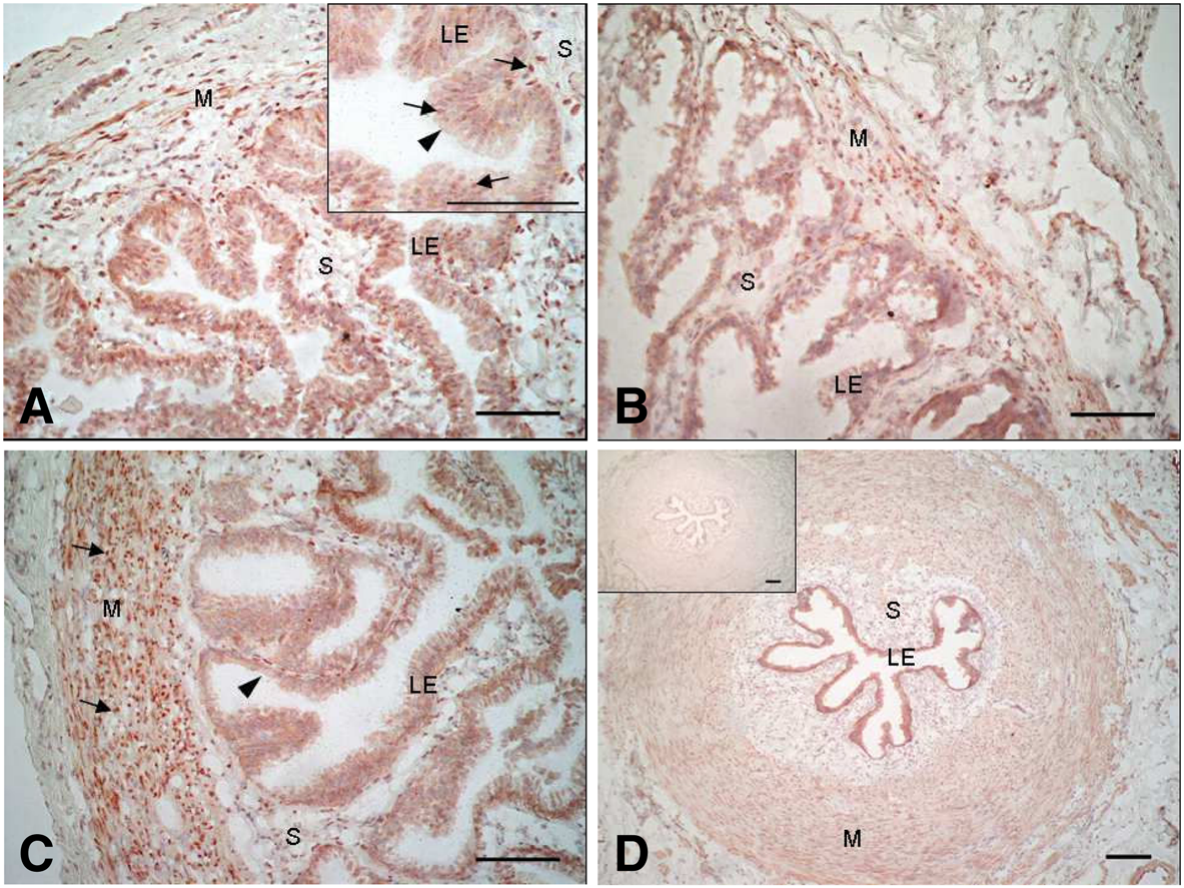
**Localisation of PR in the bovine oviduct by immunohistochemistry.** Representative images of PR immunohistochemical localisation in the ampulla (**A, B**) and the isthmus (**C, D**) of bovine oviducts at Day 1.5 (**A, C**) and Day 5 (**B, D**) post-AI. A inset: magnification of the luminal epithelium showing a nuclear and cytoplasmic staining in the epithelial cells and a nuclear staining in the stromal cells. D inset: control section incubated with mouse IgG in place of primary antibody. Black arrows indicate nuclear staining and arrow heads indicate cytoplasmic staining. LE: luminal epithelium; S: stroma; M: muscle. Original magnification A-C x200 and D x100. Scale bars: 100 μm.

**Figure 4:**
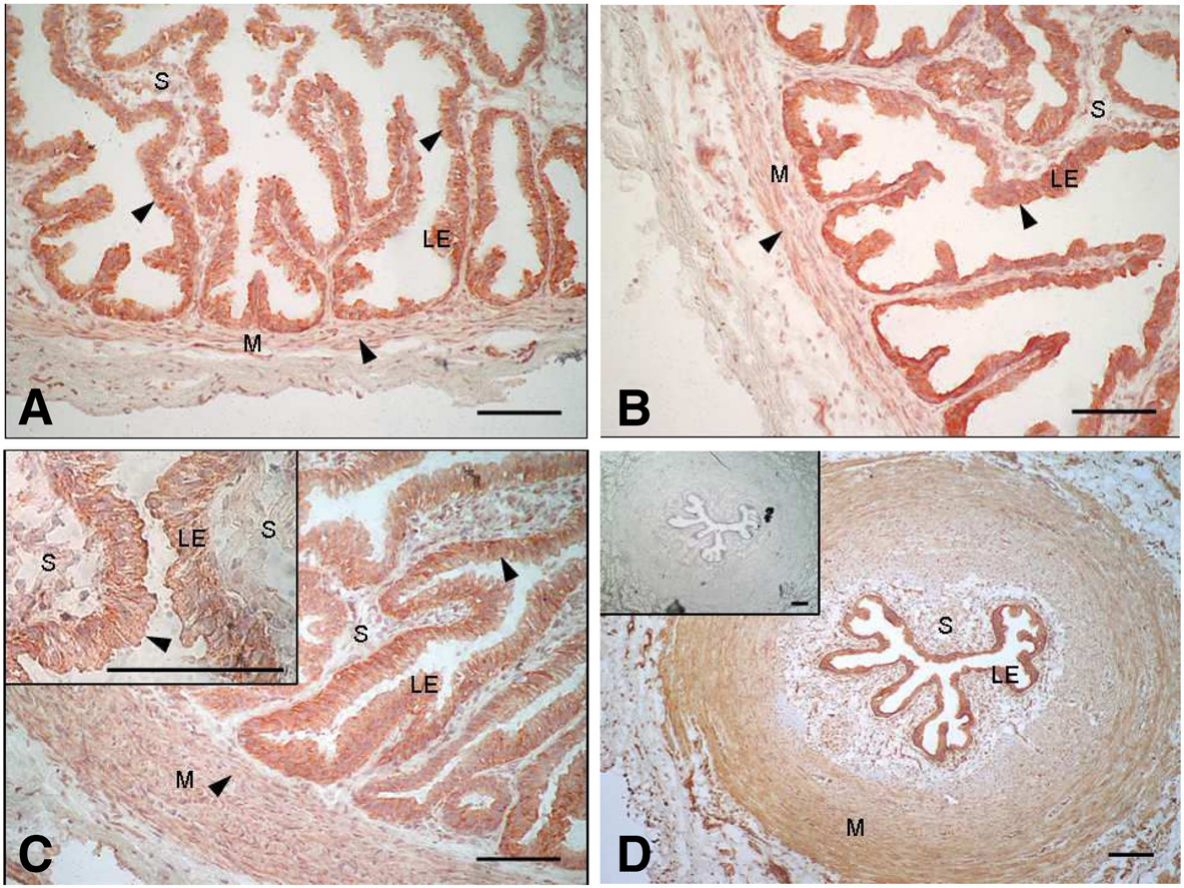
**Localisation of PGRMC1 in the bovine oviduct by immunohistochemistry.** Representative images of PGRMC1 immunohistochemical localisation in the ampulla (**A, B**) and the isthmus (**C, D**) of bovine oviducts at Day 1.5 (**A, C**) and Day 5 (**B, D**) post-AI. C inset: magnification of the luminal epithelium showing a cytoplasmic staining mainly localised in the epithelial cells. D inset: control section incubated with rabbit serum in place of primary antibody. Arrow heads indicate cytoplasmic staining. LE: luminal epithelium; S: stroma; M: muscle. Original magnification A-C x200, D x100 and C inset x1000. Scale bars: 100 μm.

**Figure 5:**
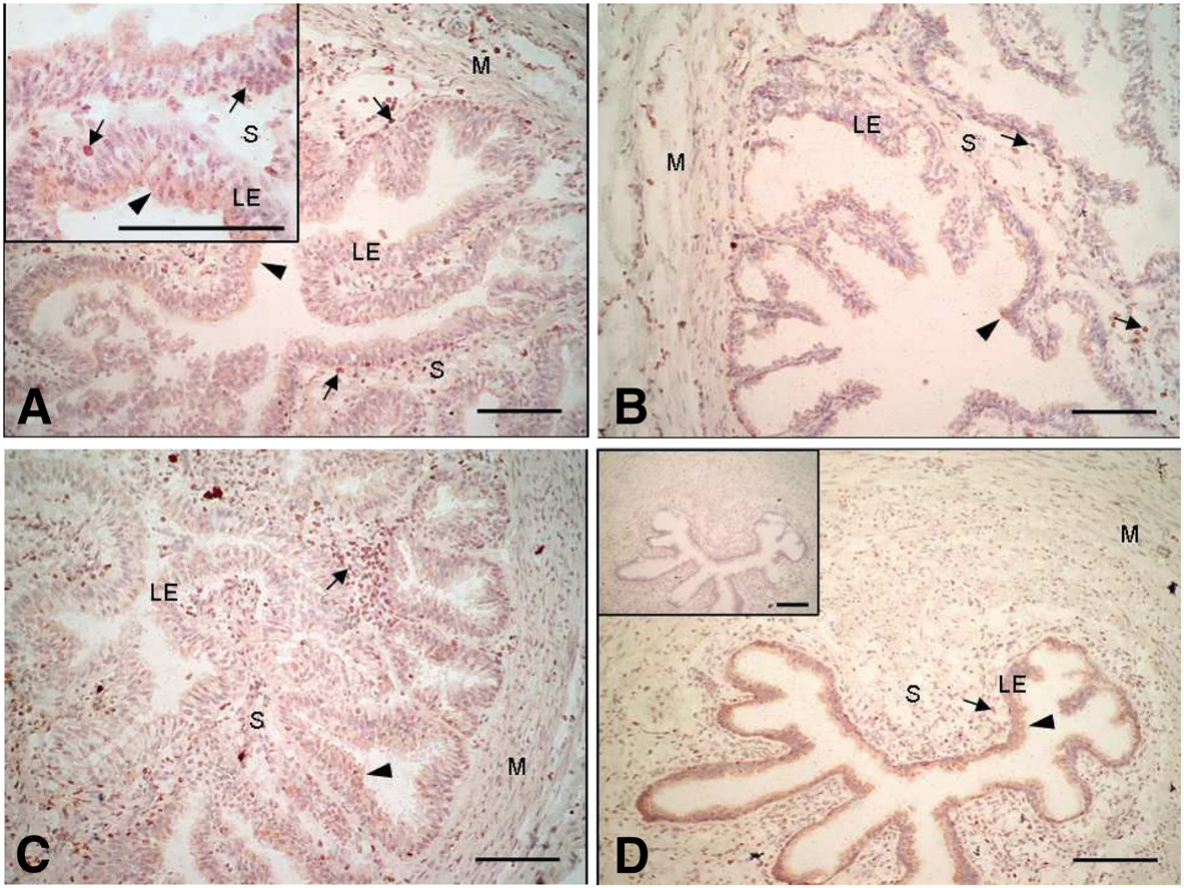
**Localisation of PGRMC2 in the bovine oviduct by immunohistochemistry.** Representative images of PGRMC2 immunohistochemical localisation in the ampulla (**A, B**) and the isthmus (**C, D**) of bovine oviducts at Day 1.5 (**A, C**) and Day 5 (**B, D**) post-AI. A inset: magnification of the luminal epithelium showing a nuclear and cytoplasmic staining of epithelial cells. D inset: control section incubated with mouse IgG1 in place of primary antibody. Black arrows indicate nuclear staining and arrow heads indicate cytoplasmic staining. LE: luminal epithelium; S: stroma; M: muscle. Original magnification A-D x200 and A inset x1000. Scale bars: 100 μm.

Immunocytochemistry for PR revealed a specific nuclear staining in the luminal epithelium, the stromal cells and the muscle layer in both the ampulla (Figures 3A,B) and the isthmus (Figures 3C,D). A weak cytoplasmic staining was also present in the luminal epithelium in both oviductal sections (Figure 3A inset, C). Examination of PGRMC1 localisation revealed an intense cytoplasmic staining in the luminal epithelium in both the ampulla (Figures 4A,B) and the isthmus (Figures 4C,D), while the muscular and stromal layers were weakly stained in both parts of the oviduct. Examination of PGRMC2 localisation revealed a cytoplasmic staining in the luminal epithelium and a nuclear staining in the stromal and muscle layers in both the ampulla (Figures 5A,B) and the isthmus (Figures 5C,D). Higher magnification showed a discrete nuclear PGRMC2 staining in the epithelial compartment (Figure 5A inset). No obvious differences in localisation patterns of PR, PGRMC1 and PGRMC2 were observed between ipsi- and contralateral oviducts or according to the stage post-ovulation (Day 1.5, Day 4 or Day 5).

### Experiment II: Expression of *PR*, *PGRMC1* and *PGRMC2* mRNAs in oviduct epithelial cells at Day 3.5 post-ovulation

**Figure 6:**
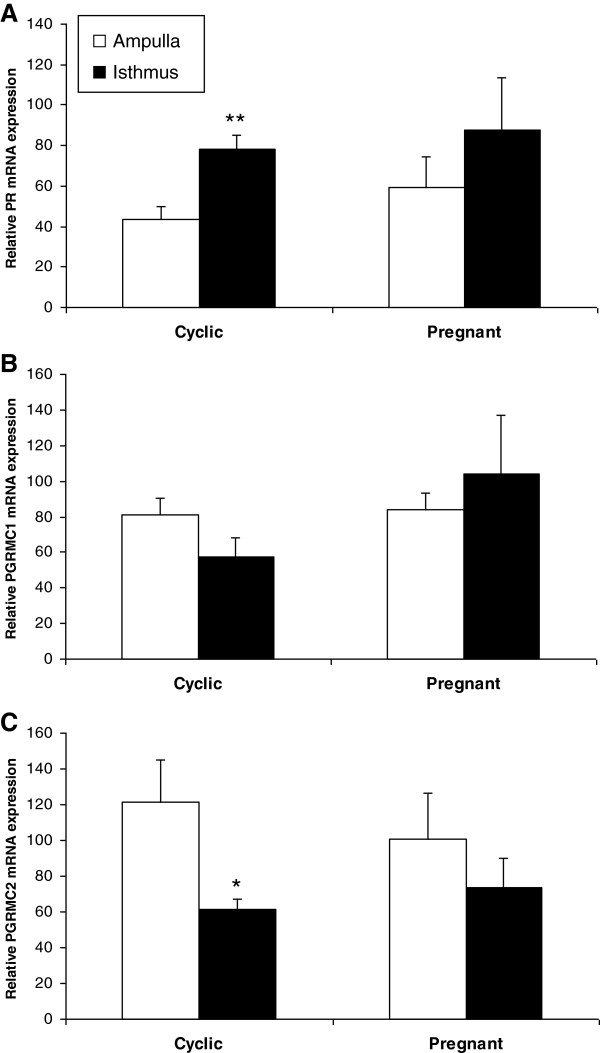
**Quantitative expression of PR, PGRMC1 and PGRMC2 mRNAs in the bovine oviduct.** Differential mRNA expression of *PR* (**A**), *PGRMC1* (**B**) and *PGRMC2* (**C**) between the ampulla and the isthmus in bovine oviduct epithelial cells at Day 3.5 post-ovulation in either cyclic or pregnant cows. Data are means ± SEM of 4 cows for each status (ipsilateral and contralateral oviducts were grouped). Concentrations of mRNAs were measured in duplicate. Means with designations (*) and (**) are significantly different at p < 0.05 and p < 0.01, respectively.

To further characterise the expression of P4 receptors, a RT-qPCR approach was applied to oviduct epithelial cells at Day 3.5 post-ovulation in both cyclic and pregnant cows. Statistical analysis revealed no effect of either the cow status (cyclic *versus* pregnant) or the side relative to the CL (ipsilateral *versus* contralateral) on any gene expression. However, when ipsilateral and contralateral oviducts were grouped, the expression of *PR* mRNA was significantly higher (1.8-fold higher, p = 0.004) in the isthmus than in the ampulla in cyclic cows (Figure 6A). In contrast, while *PGRMC1* expression did not vary according to the oviduct section (Figure 6B), the amount of *PGRMC2* mRNA was significantly higher (2-fold higher, p = 0.04) in the ampulla than in the isthmus in cyclic cows (Figure 6C). The effect of the oviduct region on *PR* and *PGRMC2* gene expression remained significant when cyclic and pregnant oviducts were pooled (p = 0.03 for both receptors).

## Discussion

Our results demonstrate the expression of PR, PGRMC1 and, for the first time, of PGRMC2, in several compartments of the bovine oviduct between Day 1.5 and Day 5 post-ovulation. To our knowledge, this is the first exploration of *PGRMC1* and *PGRMC2* gene expression in this organ. At Day 3.5 post-ovulation, the *PR* gene was more highly expressed in epithelial cells from the isthmus than from the ampulla, whereas *PGRMC2* displayed an opposite pattern of expression. However, neither the side relative to the CL nor the pregnancy status changed the gene expression of P4 receptors.

Western blot analysis of PR detected both PRA and PRB subtypes in oviduct samples, as previously reported in bovine epithelial cells [18]. Although both PRA and PRB bind P4, several studies have shown that, depending on the cell and target genes, PRA and PRB have remarkably different transcriptional activities [31]. However, little information is available so far with respect to the respective roles of PRA and PRB in mediating P4 action in the mammalian oviduct [32,33]. Nevertheless, in the present study, nuclear P4 receptors were expressed between Day 1.5 and Day 5 post-ovulation in both the ampulla and the isthmus. These results are in keeping with earlier reports on PR expression in bovine whole-oviduct sections [17,34] and in isolated oviduct epithelial cells [18]. A nuclear immunostaining was observed in the luminal epithelial cell layer, as well as in the muscle layer and stromal tissue, in agreement with earlier reports using the same anti-PR antibody [18] or some others [17,34,35] in bovine oviduct samples collected during the first five days of the oestrous cycle. Accordingly, one of the latter studies reported that the muscle layer was positively stained only during the early luteal phase (days 1–5) compared with other phases of the oestrous cycle in the cow [18].

We observed that PR, PGRMC1 and PGRMC2 were expressed in both the ampulla and isthmus at all stages examined. To our knowledge, this is the first report of PGRMC2 expression in the mammalian oviduct. Western blot analysis for PGRMC2 revealed one band at 26 kDa, as previously described in bovine cumulus and luteal cells [23]. In contrast, two signals at 23 kDa and 28 kDa were observed on PGRMC1 western blots. While the PGRMC1 protein is usually detected as a single signal between 24 and 28 kDa [21,23,25,26], multiple bands were previously reported at the same apparent molecular weights in murine placental tissues [36], which were assumed to represent PGRMC1 oligomers [20]. Various protocols of sample fixation/preparation were used in the latter and present studies and could explain these discrepancies. These conflicting findings could be also attributed to the different tissues/cells and subcellular compartments analysed. Indeed, an antibody from the same source was reported to detect a single band in membrane preparations from human and rat granulosa/luteal cells [21,37] and multiple bands in whole murine uterine tissues [36].

PGRMC1 has been reported in various subcellular locations, including plasma membrane [21,38], cytosol [36] and nucleus [26,36,37]. In this study, PGRMC1 was mainly localised in the cytosol of luminal epithelial cells, and to a lesser extent in muscular and stromal cells. This differs from findings in a previous study [26] that localised PGRMC1 in the bovine oviduct: the protein was observed in both the nucleus and cytoplasm of the luminal epithelium, endothelium and muscle layer at equivalent staining levels. This divergence may be due to differences in stages of estrous cycle examined, which were not precisely timed in the latter study [26], as well as to differences in antibodies and protocols used. The fact that we had a clear signal in the nucleus for PR and PGRMC2 demonstrates, however, the efficacy of the method used. Furthermore, our results are in keeping with those of Aparicio *et al.*, [23], who localised PGRMC1 only in the cytoplasm of bovine cumulus cells. With the antibody used in the present study, the immunostaining for PGRMC1 was intense in the nucleus of human and rat granulosa/luteal cells [21,37,39]. However, the subcellular localization of PGRMC1 was reported to change dramatically after eCG treatment in the immature rat ovary [21] and depending on the pregnancy stage in the mouse uterus [36]. Thus, the expression of PGRMC1 in various tissues and subcellular compartments of the bovine oviduct might be regulated by the hormonal environment and requires further investigations. The mRNA and/or protein for PGRMC2 have been detected previously in the murine [36] and macaque [40] endometrium, human choriodecidua [41], newborn rat ovary [42], and bovine cumulus cells [23]. Various subcellular localisations for the PGRMC2 protein were previously observed depending on the species and cell tissue: throughout the cytoplasm in bovine cumulus cells [23], in the cytoplasm of the luminal and glandular epithelia and in the nuclei of the stromal cells in the macaque endometrium [40].

Oviducts in the present study were collected from cows treated for estrous synchronisation. Although not well documented, compared with natural cycles, synchronisation protocols may have some impact on the oviduct physiology. Furthermore, oviduct epithelial cells from pregnant animals in the present study were collected from cows inseminated twice at Day 0 and Day 0.5 post-ovulation. Although the lumen of the oviducts was rinsed prior to their use for RNA extraction, sperm cells attached to the luminal epithelium, especially in the isthmic sperm reservoir [43], may have been picked up in these extraction processes. Since PR and also PGRMC1 and PGRMC2 have been identified in porcine [30] and human [22] sperm, one may question whether our results include expression data from bovine sperm cells. Several sets of data seem to exclude this hypothesis. First, the number of spermatozoa counted per entire bovine oviduct between 17 and 20 h after insemination is around a few tens [44]. Second, nuclei from spermatozoa that transit in the female genital tract are transcriptionally inactive [45] and therefore, mRNAs from spermatozoa have no chance of being co-amplified during the RT-qPCR performed on oviduct samples. Thus, it appears reasonable to exclude possible contamination with bull spermatozoa in our expression data.

While the gene expression of *PR* in target tissues is classically up-regulated by estradiol and down-regulated by P4 [31], the factors that regulate the expression of *PGRMC1* and *PGRMC2* are largely unknown. Nonetheless, P4 has been identified as a good candidate for the regulation of *PGRMC1* gene expression in mouse brain and placenta [36,38] as well as in the endometrium of human [19,46,47] and rhesus monkey [48]. Furthermore, the gene expression of both *PGRMC1* and *PGRMC2* was recently demonstrated in the canine oviduct, in which their mRNA levels varied significantly with the stage of the estrous cycle [49]. The bovine oviduct ipsilateral to the CL has been shown to contain higher levels of P4 compared with the contralateral oviduct during the luteal phase of the estrous cycle [50]. However, in this study, quantitative RT-PCR applied on oviduct epithelial cells did not reveal any significant difference in the gene expression of *PRPGRMC1* or *PGRMC2* between ipsilateral and contralateral oviducts. Accordingly, Rottmayer *et al*. [51] reported no difference in *PR* mRNA expression between epithelial cells collected from bovine ipsilateral and contralateral oviducts. By a transcriptomic approach, 35 genes were found to be differentially expressed between epithelial cells from ipsilateral *versus* contralateral bovine oviducts collected at Day 3.5 post-oestrus [52]. However, in accordance with our findings, genes for P4 receptors were not among the differentially expressed genes. Furthermore, the localisation pattern of the three P4 receptors observed in this study did not reveal any difference between the two oviducts. In accordance, PGRMC1 was immunolocalised by others in a similar pattern in both oviducts from cyclic cows [26]. In this study, the effect of the pregnancy status on the gene expression of nuclear and membrane P4 receptors was explored for the first time in the bovine oviduct. Although plasma P4 concentrations do not differ between cyclic and pregnant cows at Day 3.5 post-ovulation [53], local differences in tubal vascularisation and ciliary beating in the bovine oviduct epithelium were previously shown to depend on the presence or absence of an embryo [8,9]. However, here, the pregnancy status did not influence the expression of any of the P4 receptors at Day 3.5 post-ovulation. Similarly, in the bovine endometrium, the level of PR mRNAs was not influenced by the pregnancy status during the early post-ovulation period (Days 5–7) [54]. Our results do not exclude differential expression of these receptors between cyclic and pregnant cows in other oviductal tissue layers or at later stages post-ovulation. Indeed, in the bovine endometrium, *PR* mRNA concentration was lower in pregnant cows than in cyclic cows at Day 13 post-ovulation, but not earlier [54].

In the present study, the *PR* gene was more highly expressed in epithelial cells from the isthmus than from the ampulla, whereas *PGRMC2* displayed an opposite pattern of expression. Higher amounts of *PR* mRNAs in the isthmus compared with the ampulla were previously reported in the bovine [18,34] and rat [32] oviduct luminal epithelia. However, in the pig oviduct, *PR* mRNAs were measured at comparable levels in both oviduct regions [55]. As the ampulla is mainly implicated in oocyte transport and fertilisation [7], a positive effect of PGRMC2 in these tubal events might be assumed. Furthermore, a higher expression of PR in the isthmus might have beneficial effects in the specific functions of this part of the oviduct, i.e. in the storage of spermatozoa before fertilisation and/or in the timed transport of the embryo toward the utero-tubal junction [7]. The factors that could regulate the differential gene expression of *PR* and *PGRMC2* in the two parts of the bovine oviduct are still unknown. Previous measurement of P4 levels in whole-oviduct sections collected during the first 5 days post-ovulation revealed no difference in P4 concentrations between the ampulla and isthmus [50]. Thus, it seems likely that factors other than local P4 regulate the region-specific gene expression of *PR* and *PGRMC2* in the oviduct epithelium.

An important question remains the potential roles of nuclear and membrane P4 receptors in the regulation of tubal functions. A role for PGRMC1 in the mediation of the anti-apoptotic effects of P4 has been reported in ovarian granulosa cells [19-21,56]. Furthermore, PGRMC1 has been recently implicated in the mediation of myometrium relaxation during pregnancy in human [25]. Of interest, a proteomic approach showed that PGRMC2 was up-regulated in the human choriodecidua during term and preterm labor [41]. A central role of the oviduct during the post-ovulation period is to allow the transport of the cumulus-oocyte complex to the site of fertilisation, then of the early embryo toward the uterus, achieved by synchronised ciliary beating, smooth muscle contraction and flow of tubal secretions [14]. It might be assumed that both PGRMC1 and PGRMC2 are involved in tubal contraction and relaxation. However, as both receptors were expressed in the luminal epithelium, the mediation of P4 action on tubal ciliary beating or fluid secretion cannot be excluded. Further experiments would be required to determine the potential functions of P4 nuclear and membrane receptors in the bovine oviduct.

## Conclusions

The present study confirms the expression of PR and PGRMC1 and demonstrates for the first time the expression of PGRMC2 in several compartments of the bovine oviduct. Furthermore, the expression of *PR* and *PGRMC2* mRNAs in oviduct epithelial cells at Day 3.5 post-ovulation varied with the oviduct region. These results suggest that P4 regulates the tubal micro-environment in the early post-ovulation period in the cow and mediates its actions through classical as well as non classical pathways.

## Abbreviations

AI, Artificial insemination;CL, Corpus luteum; mRNA, Messenger ribonucleic acid;P4, Progesterone;PGRMC, Progesterone receptor membrane component;PR, Progesterone receptor;qPCR, Quantitative real-time polymerase chain reaction;RT-PCR, Reverse transcript-polymerase chain reaction.

## Competing interests

The authors declare that they have no competing interests.

## Authors’ contributions

MSD designed and conducted experiment, analysed data and drafted the paper; OS designed and conducted experiment, analysed data and revised the manuscript; SP conducted experiment and analysed data; MC conducted experiment and analysed data; FC designed and conducted experiment, analysed data and revised the manuscript. All authors read and approved the final manuscript.
